# Petrology and Au-PGE investigation dataset of the recent alluvial sediments from the Ngaye River watershed, northern Cameroon

**DOI:** 10.1016/j.dib.2023.109567

**Published:** 2023-09-13

**Authors:** Paul-Desire Ndjigui, Estelle Huguette O. Ngono, Soureiyatou Fadil-Djenabou

**Affiliations:** a Department of Earth Sciences, University of Yaoundé I, P.O. Box 812, Yaoundé, Cameroon; b Department of Live and Earth Sciences, Higher Teacher's Training College, University of Maroua, P.O. Box 55, Maroua, Cameroon

**Keywords:** Clastic sediments, Northern cameroon, Petrological data, Au-PGE investigation

## Abstract

Petrological data of the recent alluvial sediments from the Ngaye River watershed in the Northern Cameroon were used in order to infer their origin with the probable source rocks as gneisses, amphibolites, and granites; and to investigate the occurrence of precious metals like Gold and Platinum Group Elements (Au-PGE). The Ngaye River watershed is located in the Adamawa plateau from the Adamawa-Yade Domain (AYD) in the Central Cameroon, Central Africa. This region is characterized by two contrasted seasons which induce a savanna vegetation cover with gallery forests along the rivers. The field works were done during the dry season. During this season, several whole fractions of sediments were sampled at six vertical lithological sequences along the right terrace of the Ngaye Rivers. The selection of samples was linked to their grain size and color. To better understand the origin of sediments, twenty-one rock samples (gneisses, amphibolites, and granites) were also collected. The grain size distribution was done using the Robinson's standard Pipetting Method. Thin sections were done for the determination of mineral assemblages and textural features of rocks using Leica DM 750P optic microscope. Mineralogical compositions of sediments were obtained using the X-ray diffraction (XRD) instrument. The major elements data were determined using the X-ray Fluorescence spectrometry (XRF). The Inductively Coupled Plasma-Mass Spectrometry (ICP-MS) was used to obtain the concentrations of trace elements including rare earth elements and Au-PGE. The investigation of Au-PGE was done only in amphibolites as well as in the concentrate heavy minerals (160–100 µm) from sediments of lithological sequence A. Data of this paper are further presented and discussed in Ndjigui et al. [1].

Specifications Table

**Table:** 

Subject	Earth Sciences
Specific subject area	Petrology and Mining Geology
Type of data	Table and figures
How the data were acquired	Field investigations: GPS map (Garmin 62S); sampling; The color of sediments: Munsell Soil Color Book; Making of thin sections of rocks: Paris School of Mines (France); The grain size distribution in the bulk fractions of sediments: Robinson's standard pipetting Method; Microscopic observations: Leica DM 750P microscope (University of Yaoundé I, Cameroon); Heavy minerals observations: binocular magnifying glass (University of Yaoundé I, Cameroon); Mineral phases: heavy mineral fractions (University of Yaoundé I) and X-Ray powder diffraction (Geoscience Laboratories (Sudbury, Canada)); Major elements: RIX-3000 wavelength-dispersive X-Ray Fluorescence spectrometer (Geoscience Laboratories, Sudbury, Canada); Trace elements (including rare earth elements): Inductively Coupled Plasma-Mass Spectrometry (Geoscience Laboratories (Sudbury, Canada)); Investigation of Au-PGE in amphibolites and concentrate heavy minerals (160–100 µm): Nickel-Sulfur Fire Assay Method followed by Te-coprecipitation (NFS-FA) (University of Quebec at Chicoutimi (UQAC, Canada). Binary and ternary diagrams were done to enhance their distribution.
Data format	Raw and analyzed.
Description of data collection	Two criteria were used to select sediment samples: field texture and color. Seventeen (17) bulk fractions of sediments from six lithological sequences (MD (sequence A: 200 cm of thickness), NGY (sequence B: 160 cm), NGA (sequence C: 135 cm), NGB (sequence D: 160 cm), NGE (sequence E: 120 cm) and NGF (sequence F: 70 cm)) were collected (Fig. 1). For probable source rocks, only twenty-one (21) fresh samples were collected in the outcrops of the Ngaye catchment, seven by lithotype (gneisses, amphibolites, and granites). Ten (10) samples were selected for thin section preparation. All samples (sediments and probable source rocks) were conducted to laboratories for analysis. The collecting of samples was done using hammer for rock samples, and a small peel for clastic loose sediments. Loose samples were packed. Each sample was codified linked to the sample location (MD, NGB, NGLA, NGE, NGF, and NGY). About hundred kilograms were collected for each sample.
Data source location	The Ngaye watershed is located in Northern Cameroon (Cameroon, Central Africa) as reported in [1]. Data were collected in the Adamawa-Yade Domain (AYD) of the Central African Fold Belt (CAFB). Data concerning source location (latitude and longitude using GPS) were also presented in Table 1.
Data accessibility	Repository name: Mendeley data Doi:10.17632/298y366ybd.4. Direct URL to data: https://data.mendeley.com/datasets/298y366ybd/4
Related research article	Paul-Desire Ndjigui, Elie Constantin Bayiga, Vincent Laurent Onana, Soureiyatou Fadil-Djenabou, Gaelle Sandra Assomo Ngono. Mineralogy and geochemistry of recent alluvial sediments from the Ngaye River watershed, northern Cameroon: Implications for the surface processes and Au-PGE distribution. *Journal of African Earth Sciences* 150 (2019), 136-157 [1].

## 1 Value of the data

• The dataset can be used to understand the origin of sediments as well as the effect of climate variability during paleoweathering, erosion, transportation and deposit.
• The dataset could be also used to better recognize the extension of the Cameroon Volcanic Line (CVL) in the Adamawa-Yade Domain (AYD) linked to the presence of olivine, characteristic mineral of basalts.
• The dataset could be used for the petrological characterization of sediments under tropical climate.
• The dataset highlight the moderate weathering of source rocks, specific to the tropical climate with contrasted seasons.
• The dataset could be also used as guide to the future investigation of precious metals in mafic rocks (e.g.: amphibolites) as well as in sediments derived from the same mafic rocks.

## 2 Objective

The article is focused on the detailed methodology used in the related research article [1]. This dataset is based on the mineralogy and geochemistry of the recent sediments from Ngaye watershed. It aims to determine from geological features of sediments in order to infer the nature of source rocks and mining surveys of Gold and Platinum Group Elements (Au-PGE). The impact of the weathering processes of source rocks is assessed from the weathering indexes and alpha indices using some chemical elements such as Ba, Ca, Mg, K, Sr, and Sb. Moreover, the behavior of chemical elements in these sediments could be used for the precious metals prospection. This article presents data that are not included in the related research article [1].

## 3 Data Description

**Fig. 1:**
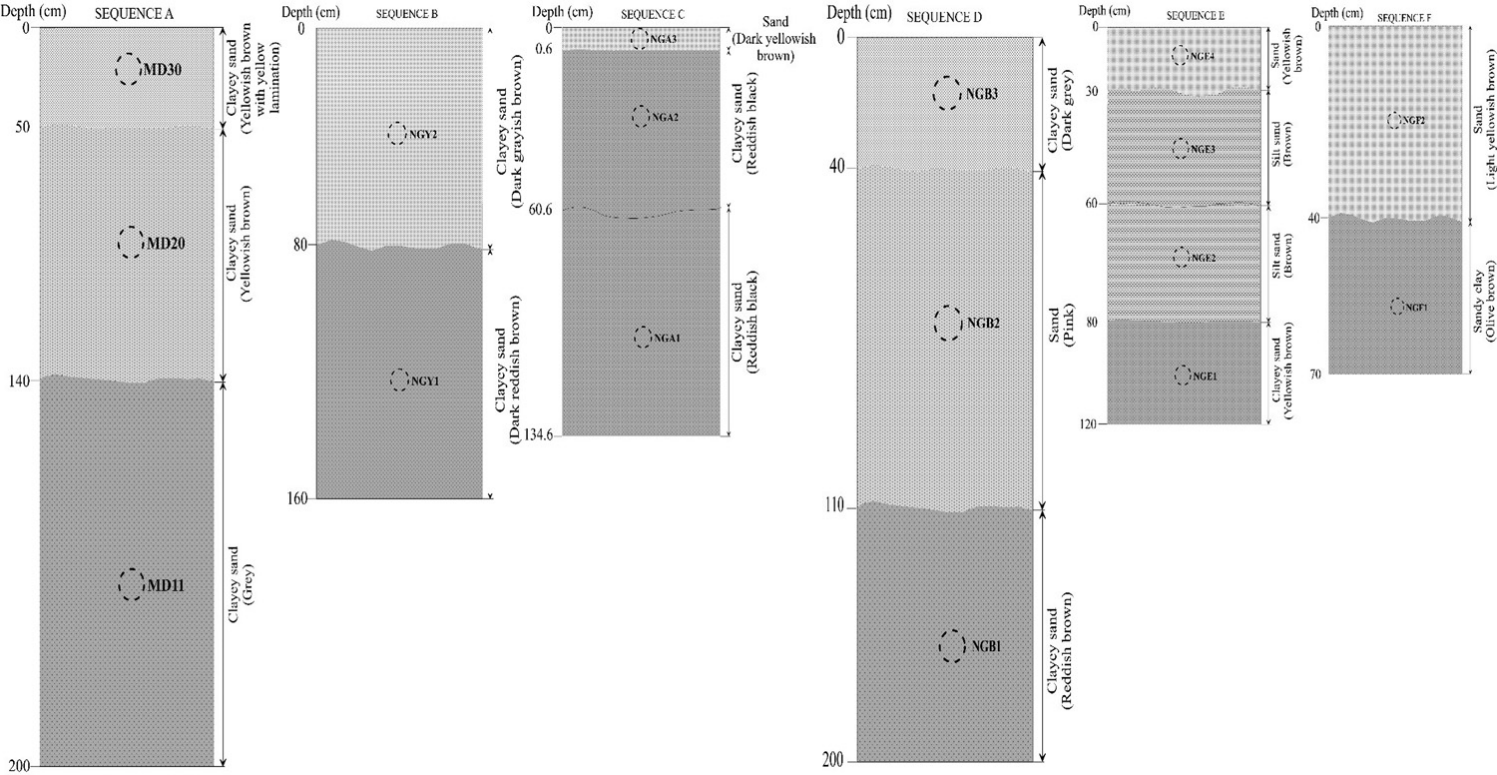
Sample location in different lithological sequences.

**Fig. 2:**
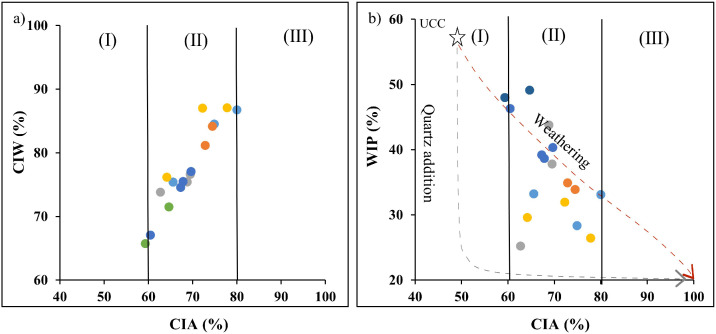
Binary diagrams illustrating the moderate weathering of source rocks: a) CIW vs. CIA; b) WIP vs. CIA.

**Fig. 3:**
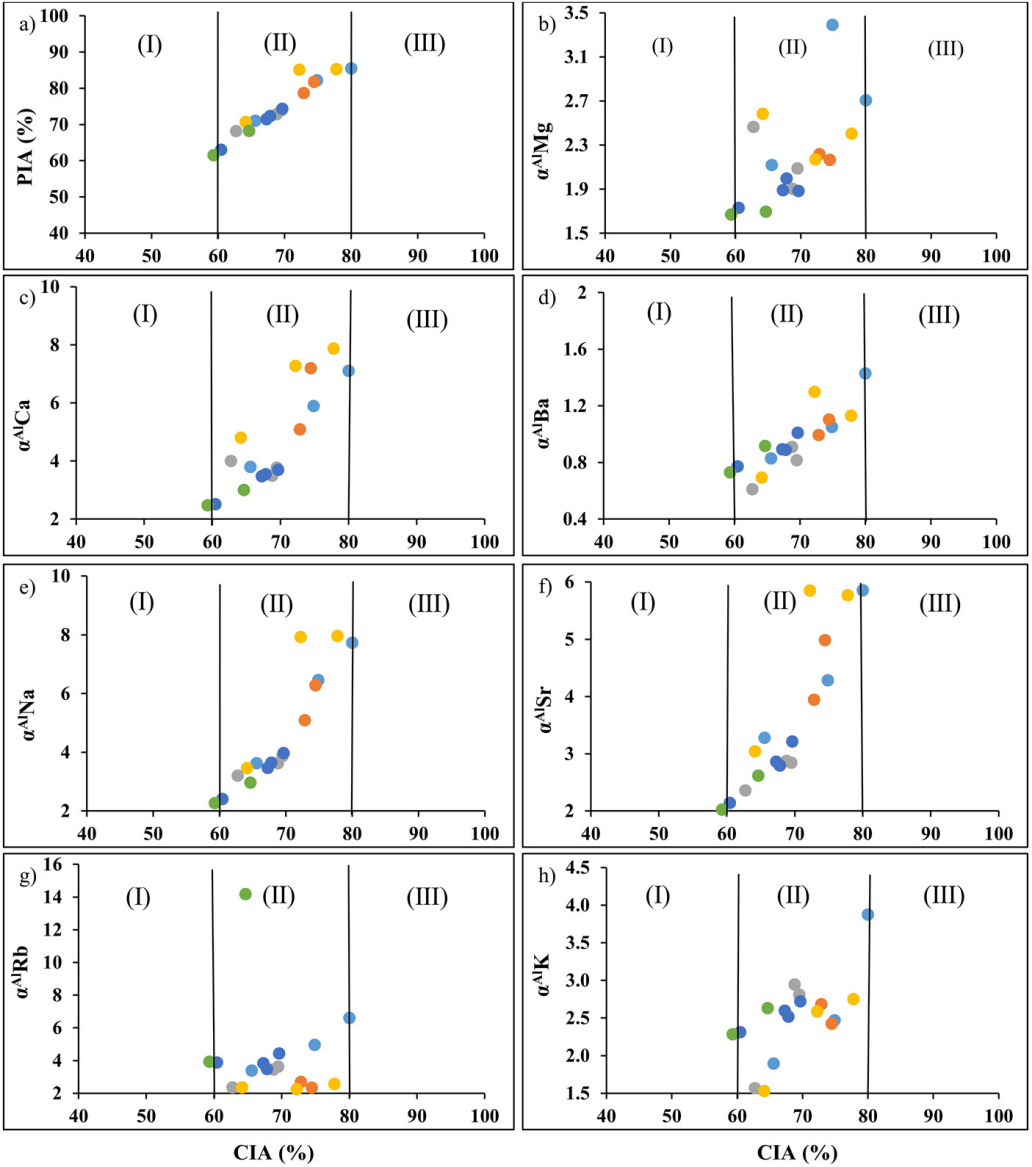
Binary diagrams illustrating the moderate weathering of source rocks using weathering and alpha indices: a) PIA vs. CIA; b) α^Al^Mg vs. CIA; c) α^Al^Ca vs. CIA; d) α^Al^Ba vs. CIA; e) α^Al^Na vs. CIA; f) α^Al^Sr vs. CIA; g) α^Al^K vs. CIA; h) α^Al^Rb vs. CIA.

Seventeen (17) samples were collected in different points of the right terraces of the Ngaye Rivers. Twenty-one (21) rock samples were taken in the outcrops of the Ngaye catchment. All these samples were pre-treated. Alluvial sediments were collected after field observations. All these samples were located in six lithological sequences. The criteria of description are the color, the texture and the thickness of each layer. All these features differ from one layer to another. The color variation, the thickness and the texture within the different sequences reflect the conditions of alluvial material deposit (Table 1; Fig. 1). The filed texture was confirmed in the laboratory using the Robinson's pipetting method. These materials have clay, sandy clay, silty sand or sand textures. An aliquot fraction of samples was used to the extraction of heavy minerals used dense liquid. The heavy minerals study confirms the nature of source rocks. The mineral assemblage of sediments was obtained using the XRD technique (Table i). This mineral assemblage refers to the variety of lithology (granites, gneisses, amphibolites and accessory basalts). The felsic contribution is confirmed by chemical data carried out using a quality control standard (Tables ii and iii). The concentrations of major elements were determined using the X-ray diffraction (XRF) technique (Table iv). The determination of trace elements including rare earth elements was done using the Inductively Coupled Plasma-Mass Spectrometry (ICP-MS) method (Table v). The Au-PGE contents were accessed using the Nickel-Sulfur Fire Assay (NiS-FA) method by ICP-MS. The estimation of weathering intensity of source rocks was done using weathering indexes and alpha indices (Fig. 2, Fig. 3; Table vi). The overall analytical uncertainty is less than 3% for major elements and 5% for trace elements. Detailed data can be found in the repertory (https://data.mendeley.com/datasets/298y366ybd/4).

**Table 1:** Location, depth (cm) and color data of samples.

	Sequence A			Sequence B		Sequence C			Sequence D			Sequence E				Sequence F	
Longitude (E)	14°59′46′'			14°59′31′'		14°58′48′'			14°59′06′'			15°00′07′'				15°00′18′'	
Latitude (N)	7°11′24′'			7°11′31′'		7°11′35′'			7°11′53′'			7°11′06′'				7°11′02′'	
Samples	MD11	MD20	MD30	NGY1	NGY2	NGA1	NGA2	NGA3	NGB1	NGB2	NGB3	NGE1	NGE2	NGE3	NGE4	NGF1	NGF2
Thickness (cm)	160	90	50	80	80	74	60	0.6	50	70	40	40	20	30	30	30	40
Color	Grey	Yellowish brown	Yellowish brown with yellow lamination	Dark reddish brown	Dark grayish brown	Reddish black	Reddish black	Dark yellowish brown	Reddish brown	Pink	Dark grey	Yellowish brown	Brown	Brown	Yellowish brown	Olive brown	Light Yellowish brown

## 4 Experimental Design, Materials and Methods

### 4.1 Experimental design

Samples were collected in several outcrops for whole rocks and unconsolidated sediments were sampled in six lithological sequences in the alluvial terrace during the dry season (March/2009, January/2010, March/2011, and March/2013). Sediments were described, packed, and also identified by a code (e.g.: MD20, MD03, and MD05). Probable source rocks were also described and identified by a code.

### 4.2 Materials

Ngaye region is located in the Adamawa-Yade Domain (AYD) of the Central African Fold Belt (CAFB). Three types of probable source rocks occur in the Ngaye River catchment: gneisses and amphibolites intruded by granites. The presence of olivine is linked to that of basalts from the Cameroon Volcanic Line (CVL) in the Adamawa plateau. The thicknesses of sedimentary sequences vary from 70 to 200 cm. These sequences have two, three or four layers (Fig. 1).

### 4.3 Methods

Rock samples were subdivided in two blocks: the first block was used to the fabrication of the thin sections, and the second (just one kg) was air-dried, crushed and powdered at 250 µm for mineralogical and geochemical analyses. For the sediments, three fractions were used: (i) the first fraction (40 g) was send to the grain size distribution analyses; the second aliquot (∼ 100 g) was also air-dried, crushed, powdered and ended for mineralogical and geochemical analysis; and (iii) the last fraction (10 g) is purpose to the extraction of heavy minerals.

Particle size analysis, involves the determination of the percentage of elementary mineral particles (sand, silt and clay), was done in the Department of Earth Sciences (University of Yaoundé I, Cameroon). This is carried following the Robinson's standard pipetting method. The procedure is summarized in eight steps: (i) 20 g of dried sediment was withdrawn in a beaker; (ii) 10 ml of H_2_O_2_ was added in order to destroy the organic matter; (iii) 20 ml of sodium hexametaphosphate was added and passed through a mechanic agitator during two hours just to disperse completely the mineral particles (sand, silt, and clay); (iv) solution was transferred into containers and distilled water was added until 1000 ml; (v) 20 ml were withdrawn using pipet after 46 s (mass of silt and clay), 4 min 48 s (mass of fine silt and clay), and 8 hours (mass of clay only); (vi) the remaining solution with sand is being washed and rinsed, until a clear water is obtained above the surface of the sand; (vii) after dying in an oven at 105°C for 24 hours, the sand was sieved using a sieve of 200 µm in order to separate coarse sand (above 50 µm) and fine sand; and (viii) at the same time, the pipped solutions were placed at the oven at 40°C for drying and weighed, prior knowing the weight of the dishes, of each dry sample, following a set of calculations, the percentage of each particle fraction was determined.

The extraction of heavy minerals was done in the same department of the University of Yaoundé I. Heavy minerals were concentrated using bromoform. An aliquot of heavy minerals was mounted on glass slides for identification under a binocular microscope. Another quantity was affected to the determination of Au-PGE contents.

Mineralogical and geochemical analyses (major and trace elements) were carried out at the Geoscience Laboratories (Sudbury, Canada). The mineralogical instrument at Geoscience Laboratories (Sudbury, Canada) is the PAN Analytical X'PERT PRO diffractometer equipped with a monochromator. Powders were pulverized with an agate mortar and pestle (2 or 3 g) and smear mounts were prepared background silicon disks for analysis. Kα2 peaks are shown as dashed lines and do not have a “V-shaped” indicator at the top of the pattern windows. Samples were analysed with Co radiation at 40 kV and 45 mA. The following parameters were used in the X'Pert High Score Plus software for the peak identification: Minimum significance: 1.00; Minimum tip width (°2θ):0.01; Maximum tip width (°2θ): 1.00; Peak base with (°2θ): 2.00; Method: Top of smoothed peak. These data were reported under the certificate number CRT-11-0462-01 of 2.15.2012 (Ref#11-0462), CRT-12-0585-04 of 6.4.2013 and CRT-12-0585-02 from 5.7.2013 (Ref#12-0585), and CRT-14-0302-04 from 11.12.2014 (Ref#14-0302). All the QC certificates and raw data (XRD-100) are available as supplementary material files (https://data.mendeley.com/datasets/298y366ybd/4: Table i).

The determination of major and trace elements concentrations was done at the Geoscience Laboratories, Sudbury (Canada). About two grams of sample were analyzed by X-Ray Fluorescence Spectrometry using a PANalytical PW2400 wavelength dispersive spectrometry. This analyses access to the determination of the contents in major elements such as SiO_2_, TiO_2_, Al_2_O_3_, CaO, Fe_2_O_3_, MgO, MnO, Na_2_O, K_2_O, Cr_2_O_3_, NiO and P_2_O_5_. The loss on ignition (LOI) was done at 105°C under nitrogen to drive off adsorbed water and at 1000°C to eliminate volatiles and oxidize Fe. Several InHouse and international standards were used and available in Table ii (https://data.mendeley.com/datasets/298y366ybd/4).

The contents of trace elements (including rare earth elements) were determined using the Inductively Coupled Plasma-Mass Spectrometry (ICP-MS). An aliquot of each sample (0.25 g) was digested with an acidic mixture (HCl+HClO_4_) at 120°C during one week. After this period, the product was rinsed with dilute acid (HNO_3_) and dried. The residue was introduced in an acidic mixture (HNO_3_, HCl, and HF) at 100°C. Four series of samples were sent for geochemical analyses: Ref#10-0482 (MD20, MD21, MD22, MD23, MD24, MD30, MD31, MD32, MD33, and MD34); Ref#11-0462 (MD03, and MD05); Ref#12-0585 (NGB3, NGB1, NGLA3, NGA1, NGLA1, NGA2, NGY1, NGY2, NGB2, and NGLA2); and Ref#14-0302 (NGE3, NGE2, NGE4, NGF2, NGA3, NGE1, and NGF1). Two reference materials were used: InHouse Reference Materials [ISHT: 11-08135 (XRF-M01), 11-07788 (IMC-100), 12-09580 (XRF-01), 13-12060 (XRF-M01), 13-12278 (IMC-100), 15-16313 (XRF-M01), and 15-16145 (IMC-100)] with the QC names (NPD-1, MRB-29, OKUM-1, MRG-1, Orca-1, and AGV-2) and International Reference Materials [INTL: 11-11088 (XRF-M01), 11-10604 (IMC-100), 12-13032 (XRF-M01), 13-16492 (XRF-M01), 13-16722 (IMC-100), 15-22507 (XRF-M01), and 15-22227 (IMC-100)] with the QC names (NPD-1, BHVO-2, MRG-1, BIR-1, GSP-2, AGV-2, and BHVO-1). The Certified Ontario Reference Material (CORM) was also used. The Quality Control (QC) used also the Instrument Control for IMC-100 (INST: 11-09301, 11-09302, 11-09303) with QC names (CHECK-7), the Laboratory Duplicates [DUP: 11-15201 (XRF-M01), 11-14513 (IMC-100), 12-18177 (XRF-M01), 13-22889 (XRF-M01), 13-23215 (IMC-100), 15-31563 (XRF-M01), and 15-31258 (IMC-100); the QC name is DUP] and the Laboratory Blanks for IMC-100 [BLANK: 11-06543, 13-10431, 15-13933, and 15-31258]. The raw data of trace elements concentrations, Quality Control standards and DUP are available in Table iii (https://data.mendeley.com/datasets/298y366ybd/4). The ICP-MS instrument was a Perkin Elmer Elan 9000. XRF (XRF-M01) and ICP-MS (IMC-100) data are available in Tables iv and v (https://data.mendeley.com/datasets/298y366ybd/4).

The calculation of the intensity of weathering was done using different methods Data of Chemical Index of Alteration were obtained using the formula (CIA = 100*Al_2_O_3_/(Al_2_O_3_ + CaO* + Na_2_O + K_2_O)) according to Nesbitt and Young [2]. Values of Plagioclase Index of Alteration (PIA = [Al_2_O_3_ – K_2_O)/(Al_2_O_3_ + CaO + Na_2_O – K_2_O)]x100) from Fedo et al. [3] were provided. Other data were obtained using the Index of Compositional Variability formula (ICV = [(Fe_2_O_3_ + K_2_O + Na_2_O + CaO +MgO + MnO + TiO_2_)/Al_2_O_3_) from Cox et al. [4] and Weathering Index formula (WIP = 100x(CaO/0.7 + 2Na_2_O/0.35 + 2K_2_O/0.25 + MgO/0.9) from Parker [5]. Recent works [6] revealed that the single mobile (α^Al^E = (Al/E)_sample_/(Al/E)_UCC_) for alkali and alkaline-earth (Mg, Ca, Na, K, Rb, Sr, and Ba) can be compared to those of the non-mobile element (Al) to better understand the weathering processes. All data are available in Tables vi and vii (https://data.mendeley.com/datasets/298y366ybd/4).

## Ethics Statements

The field data presented in this article were obtained through several geological field works and did not require informed consent. We did not conduct human or animal studies.

## CRediT authorship contribution statement

**Paul-Desire Ndjigui:** Conceptualization, Project administration, Resources, Supervision, Data curation, Formal analysis, Visualization, Writing – review & editing, Validation. **Estelle Huguette O. Ngono:** Methodology, Data curation, Formal analysis, Investigation, Writing – original draft, Writing – review & editing. **Soureiyatou Fadil-Djenabou:** Methodology, Data curation, Formal analysis, Investigation, Writing – original draft.

## Data Availability

Petrology and Au-PGE investigation dataset of the recent alluvial sediments from the Ngaye River watershed, northern Cameroon (Reference data) (Earth/Chem)
